# The Potential Regulators of Amyloidogenic Pathway of APP Processing in Alzheimer’s Disease

**DOI:** 10.3390/biomedicines13071513

**Published:** 2025-06-20

**Authors:** Daria Krawczuk, Agnieszka Kulczyńska-Przybik, Barbara Mroczko

**Affiliations:** 1 Department of Neurodegeneration Diagnostics, Medical University of Bialystok, Waszyngtona 15A, 15-269 Białystok, Poland; daria.krawczuk@sd.umb.edu.pl (D.K.); agnieszka.kulczynska-przybik@umb.edu.pl (A.K.-P.); 2 Department of Biochemical Diagnostics, Medical University of Bialystok, Waszyngtona 15A, 15-269 Białystok, Poland

**Keywords:** Alzheimer’s disease, APP, BACE, furin, HIF-1α, alcadeins, PrP, α-synuclein

## Abstract

The amyloidogenic processing of amyloid precursor protein (APP) plays a pivotal role in the pathogenesis of Alzheimer’s disease (AD), primarily through the generation of amyloid-beta (Aβ) peptides, which aggregate to form toxic plaques in the brain. The regulation of amyloidogenic APP processing is a complex interplay of enzymes, proteins, and signaling pathways, all of which contribute to the development and progression of Alzheimer’s disease. Understanding the intricate mechanisms and molecular players involved in APP processing substantially enhances our knowledge of Alzheimer’s disease pathology and holds promise for the development of biomarkers of ongoing pathology at the earliest stages of Alzheimer’s disease. In this review, we aimed to investigate selected factors that regulate the amyloidogenic pathway of APP processing.

## 1. Alzheimer’s Disease

Alzheimer’s disease is a progressive neurodegenerative condition and the leading cause of dementia, mainly impairing memory, cognition, and behavior. The number of people worldwide affected by this disease is predicted to triple by 2050. According to global reports, in 2020, the estimated cost of AD, including treatment and care, was approximately USD 305 billion, and this number is expected to increase up to USD 1 trillion in 2050 [1]. A minority of Alzheimer’s disease cases are associated with genetic mutations in the genes encoding APP (amyloid precursor protein), PSEN1 (presenilin 1), and PSEN2 (presenilin 2), and they are typically linked to early-onset Alzheimer’s disease (EOAD), in which symptoms generally appear before the age of 65. However, the vast majority of patients exhibit late-onset Alzheimer’s disease (LOAD), which is not hereditary, with symptoms appearing later in life. Additionally, the presence of the E4 allele in the *APOE* (apolipoprotein E) gene, occurring in 16% of the population, is considered the most important risk factor, followed by cerebrovascular diseases, diabetes, hypertension, head injuries, stress, and environmental factors [2]. The histopathological characterization of AD includes the accumulation of extracellular deposits of Aβ, in the form of amyloid plaques and the formation of intraneuronal aggregates of hyperphosphorylated tau protein, called neurofibrillary tangles (NFTs). These changes in the brain are accompanied by a progressive loss of neurons, with a corresponding impairment in neuronal function and atrophy of neurons and synapses, termed neurodegeneration [3]. The disease may be divided into three stages: mild, moderate, and severe. The most common symptom is a loss of short-term memory, and then gradually other symptoms occur, such as changes in personality and behavior, deterioration of verbal communication, impairment in visuospatial tasks, and motor dysfunction [4]. The diagnosis of AD is a very complex process based on neurological and cognitive testing, evaluation of biomarkers in the cerebrospinal fluid, imaging testing such as magnetic resonance imaging (MRI), computer tomography (CT), and positron emission tomography (PET) scans, and a thorough medical history [5]. It is essential to diagnose the disease at the earliest stage possible in order to delay the progression of symptoms and to appropriately manage the disease [6].

## 2. APP Processing

APP is a type I transmembrane protein ubiquitously expressed in many tissues, with particularly high levels in neurons. It contains a large extracellular N-terminus and a short cytosolic C-terminus. Three main isoforms of APP may be distinguished, based on their amino acid length: APP770 and APP751, which are ubiquitously expressed in nonneuronal cells; and APP695, which is predominantly expressed in neurons and considered the most toxic. The full-length APP consists of 770 residues and includes a large ectodomain, a single transmembrane region, and an intracellular domain. APP has multiple functions, such as synaptic transmission and plasticity, neurogenesis, dendritic arborization, axonogenesis, cell adhesion, proliferation, and regulation of calcium metabolism [7]. In addition, APP is essential for brain development and growth by promoting long-term potentiation (LTP) and enhancing neurite outgrowth. [8].

**Figure 1:**
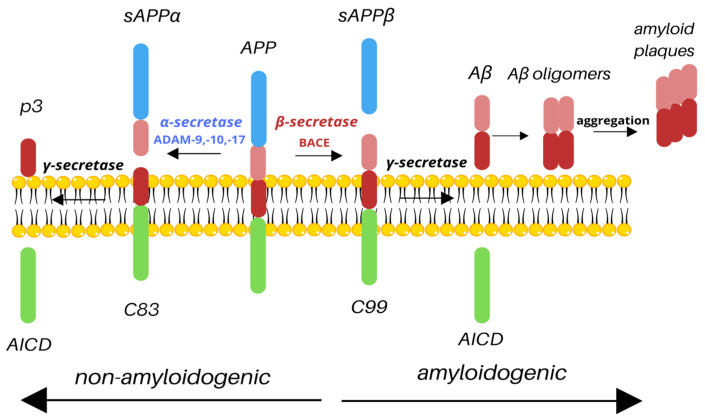
Cleavage of APP. Depending on the secretases involved, APP is processed via the non-amyloidogenic or amyloidogenic pathway. Cleavage by α-secretase (e.g., ADAM-9, -10, -17) results in the release of sAPPα and C83; C83 is then cleaved by γ-secretase to produce nontoxic p3 and AICD. However, under pathological conditions, APP may be processed by β-secretase, resulting in the production of sAPPβ and C99; C99 is then cleaved by γ-secretase. As a result, similarly to the non-amyloidogenic pathway, AICD is released; however, such cleavage also produces toxic Aβ species, which aggregate into amyloid plaques, main hallmark of Alzheimer’s disease.

APP undergoes two types of cleavage: non-amyloidogenic and amyloidogenic, based on the secretase involved. The non-amyloidogenic pathway takes place under normal physiological conditions. Cleavage by α-secretase results in releasing the N-terminal extracellular soluble APP domain (sAPPα) and a membrane-attached C83 fragment. The C83 fragment is then cleaved by γ-secretase to release the p3 outside of the cell and the amyloid precursor protein intracellular domain (AICD) inside of the cell [9]. It has been shown that during the acute brain insult, APP increases the production of sAPPα, which improves synaptic plasticity and regulation of inflammatory signaling pathways [10]. It has also been reported that the CSF concentration of sAPPα is lower in AD patients and that it correlates with cognitive dysfunction due to the degeneration of cholinergic neurons in the basal forebrain [11]. Similarly, studies showed that the reduction in CSF sAPPα is associated with AD severity [8]. The amyloidogenic processing of APP involves cleavage by the β-secretase cleaving enzyme (BACE1), resulting in the release of a large, soluble extracellular N-terminal sAPPβ and the C99. The C99 fragment is then cleaved by γ-secretase, resulting in the production of AICD, which stays inside the cell, and Aβ is released outside of the cell (Figure 1). BACE1 contains a signal peptide, a prodomain, a catalytic domain, a transmembrane domain, and a cytoplasmic domain. The signal peptide traffics the BACE1 to the endoplasmic reticulum (ER), where the cleavage of the prodomain by furin results in the production of mature BACE1 [12]. AICD, present in both pathways, is a known transcription factor with a YENPTY motif that facilitates binding to other proteins and has been shown to be one of the regulators of APP processing, promoting intracellular trafficking [7]. The final product of the amyloidogenic pathway, Aβ, consists of 37–43 amino acids, with Aβ42 isoform being the most deleterious. Aβ peptides form extracellular, soluble oligomers and plaques and insoluble fibrils, which are main hallmarks of AD [13].

While β- and γ-secretases have been central in AD research, other enzymes are gaining attention for their potential involvement in APP processing and disease progression.

Zhang et al. identified that delta-secretase (δ-secretase), also known as asparagine endopeptidase (AEP), cleaves APP at two specific asparagine residues: N373 and N585. This cleavage results in the production of APP fragments that are more prone to amyloidogenic processing, thereby increasing the generation of Aβ peptides. Moreover, δ-secretase activity increased with age in both human and mouse brains. This age-dependent activation correlated with enhanced cleavage of APP and the subsequent Aβ production. In mouse models, overexpression of δ-secretase led to an increased Aβ accumulation, synaptic dysfunction, and memory deficit. Conversely, genetic deletion or pharmacological inhibition of δ-secretase resulted in reduced Aβ levels, improved synaptic function, and better cognitive performance [14]. It has also been found that in APP/PS1 transgenic mice, AEP activity was elevated during the early stages of Alzheimer’s disease—prior to the appearance of senile plaques and cognitive decline. Furthermore, treatment with the δ-secretase inhibitor suppressed Aβ production and improved memory deficits. Thus, AEP upregulation may serve as an early marker of AD onset [15].

Studies have also identified eta-secretase (η-secretase), a protease with activity attributed primarily to the membrane-type 5 matrix metalloproteinase (MT5-MMP). Cleavage by the MT5-MMP results in the production of the eta carboxyl terminal fragment (ηCTF), which then undergoes cleavages by β- and γ-secretases to produce Aηα and Aηβ peptides. ηCTF is predominantly localized in the Golgi apparatus, endosomes, and extracellular vesicles. Aη-α, in particular, has been shown to impair synaptic plasticity and reduce LTP, a critical process for memory function [16,17]. Piłat et al. observed that the absence of MT5-MMP led to reduced neuroinflammation and prevented the reduction in dendritic spine density in 5xFAD neurons, suggesting a protective effect on synaptic function [18]. Recently, research has significantly advanced our understanding of the role of platelets in APP processing [19,20]. Contrary to neuronal APP, platelet APP is processed mainly through the non-amyloidogenic pathway by α-secretase. However, among AD patients, it was observed that the processing of APP is shifted to the amyloidogenic pathway, proving that activated platelets are implicated in the pathogenesis of AD [21]. An interesting study conducted by Ramos-Cejudo et al. aimed to investigate the relationship between platelet function and the risk of developing dementia over a 20-year period. It has been revealed that higher platelet aggregation response, measured by the response to adenosine diphosphate (ADP) and epinephrine, was associated with an elevated risk of developing dementia. Thus, different platelet phenotypes may potentially have prognostic value in terms of predicting dementia, although further studies in this field are necessary [22].

While both cleavage pathways of APP processing have been extensively studied, there are still some critical aspects that remain poorly understood. One of the main questions is what determines the activity of α- and β-secretases under physiological and pathological conditions, and what causes the shift from the non-amyloidogenic to amyloidogenic pathway. Although numerous factors can influence these processes, such as enzyme location, substrate availability, and post-translation modifications, the exact mechanisms are still unknown. It has been shown that the activity of those secretases increases with age, but the underlying mechanisms still remain unclear [23]. Furthermore, most APP studies have been focused on APP in neurons. However, it should be noted that astrocytes, oligodendrocytes, and microglia also express APP and may contribute differently to disease progression. Although neurons have been considered the main source of Aβ production, the expression levels of Aβ processing genes are comparable between neurons and oligodendrocytes [24]. Given that oligodendrocytes may substantially contribute to Aβ production, selectively targeting Aβ generation in these cells could offer a therapeutic approach for AD that minimizes the negative effects associated with BACE1 upregulation [25]. Spatiotemporal regulation of APP and secretases in these cells is still underexplored. It has been shown that APP expression is modulated throughout life. Thus, it is crucial to verify how APP membrane trafficking is altered during aging. Furthermore, the identification of proteins interacting with APP, as well as the factors influencing them, also seems to be important for comprehending the role of APP in diverse physiological and pathological states. Most findings are derived from rodent models, which may not fully capture the complexity of human APP processing. Future research should prioritize the use of human-induced pluripotent stem cell (iPSC)-derived models, single-cell transcriptomics, and spatial proteomics to map APP processing in diverse brain regions and cell types over time [26]. Deepening our knowledge and understanding of the intricacies of APP processing pathways is pivotal for developing potential therapeutic interventions for AD, such as targeting secretases or modulating APP interactions with other proteins.

Since APP is produced in large quantities and multiple alternate pathways exist for its proteolysis, some of which lead to Aβ peptide production and some of which do not, a growing body of research is investigating the regulation of APP. In this review, we focus on some factors and proteins that may play a role in amyloidogenic APP processing, and thus in the pathophysiology of AD.

## 3. Regulators of Amyloidogenic Pathway

### 3.1. Lipid Rafts

The brain is particularly rich in lipids, and disturbances in lipid homeostasis are closely linked to neurological and neurodegenerative disorders, including Alzheimer’s disease. Moreover, in the early stages of AD, alterations in fatty acids at the level of lipid rafts and cerebral lipid peroxidation were identified [27]. Lipid rafts are specialized organelle membrane microdomains rich in cholesterol and sphingolipids that take part in a variety of important biological processes, such as cell adhesion and signaling, trafficking, and protein sorting. They are characterized by combinations of sphingolipids, cholesterol, saturated fatty acids, and a reduced content of poly-unsaturated fatty acids [28]. The segregation of cholesterol and sphingolipids initially occurs in the Golgi, where nascent sphingolipids mature and are present on the plasma membrane, as well as in the membranes of the Golgi, trans-Golgi network, and endocytic compartments. Lipid rafts are thought to form different types of platforms, floating around in the liquid-disordered matrix of the cellular membranes [29]. A growing body of research indicates that the amyloidogenic processing of APP occurs in the lipid rafts. It has been shown that a significant portion of BACE1 is present in lipid rafts and that targeting the BACE1 lumenal domain to lipid rafts through the addition of a glycophosphatidylinositol (GPI) anchor increases APP processing at the β-cleavage site [30]. It has been demonstrated that increased cholesterol levels correlate with increased Aβ production and enhancement of BACE1 in lipid raft domains. Ehehalt et al. showed that cholesterol plays a key role in controlling the access of α- and β-secretases to APP. Specifically, reducing cholesterol levels decreased β-cleavage while enhancing α-cleavage. This finding indicates that the processing of APP by BACE1 is highly dependent on the lipid raft environment. In living cells, BACE1 activity relies on intact lipid rafts, whereas outside of these rafts, BACE1 seems to be inactive [31]. Other researchers analyzed the brain samples of AD patients and revealed that there is an increase in lipid ordering and lipid raft formation due to a reduction in unsaturated lipid levels. Moreover, a significant positive correlation was found between lipid viscosity and APP-BACE1 interactions [32,33].

Furthermore, APP, BACE1, and γ-secretase undergo palmitoylation, a post-translational modification that promotes their localization within lipid raft domains. S-palmitoylation is a process of reversible lipidation during which palmitoyl transferase attaches palmitate to a cysteine residue via the thioester bond, resulting in numerous effects on protein, such as its subcellular localization and trafficking, its propensity to dimerize, and partitioning between the lipid liquid-ordered (Lo) and liquid-disordered (Ld) membrane domains [34]. Protein palmitoylation influences intracellular trafficking, localization to cholesterol-rich membrane rafts, protein–protein interactions, and various signaling functions [35]. The S-palmitoylation of γ-secretase at C689 of nicastrin and C182 and C245 of APH-1 has been found to be an important factor for γ-secretase stability and raft localization [36]. Overall, the destabilization of lipid rafts observed in the early stages of AD appears to create a conducive environment for secretases to interact with APP, thereby promoting its amyloidogenic processing.

### 3.2. Alcadeins

Alcadeins (Alc), also known as calsyntenins (CSTs), are members of the cadherin superfamily, originally identified from spinal cord neurons and belonging to type I transmembrane proteins, with extracellular domains containing two cadherin repeats and an LNS (laminin α, neurexin, and sex hormone-binding globulin). Three types of alcadeins may be distinguished: α (Alcα), β (Alcβ), and γ (Alcγ), encoded by separate genes. Alcα is known to take part in membrane trafficking as a cargo receptor of kinesin-1. Alcβ acts as a synaptic adhesion molecule, and Alcγ is implicated in memory performance and cognition [37]. Alcs are primarily cleaved by APP α-secretase to generate membrane-associated Alc CTF, which is then cleaved by γ-secretase, like APP, to secrete a short peptide p3-Alcα into the cell media or the CSF, along with the liberation of the intracellular cytoplasmic domain fragment—Alcα ICD. By the association with the neural adaptor protein X11-like (X11L), Alcα and APP form a ternary complex that suppresses the cleavage of both Alcα and APP by regulation of transport of these proteins into the late secretory pathway where secretases are active. The formation of such a complex also regulates the entry of APP into lipid rafts where β-secretase is active [38,39].

It has been shown that Alcα-deficient (Alcα-KO) mice intensified brain Aβ accumulation and increased the amyloidogenic cleavage of APP through the aging process. In contrast, Alcβ-deficient mice neither affected APP metabolism nor Aβ accumulation at any age. Thus, Alcα may be neuroprotective, by suppressing the amyloidogenic cleavage of APP by β-secretase, which is distinct from Alcβ, in which p3-Alcβ peptides restore the viability in neurons damaged by toxic Aβ species [40]. Hata et al. found that the CSF p3-Alcβ37 levels were significantly reduced in AD patients compared to controls, especially at an early stage of the disease. Furthermore, the authors aimed to assess whether p3-Alc peptides were as neurotoxic as Aβ. Interestingly, mouse primary neurons, after being cultured for 24h with p3-Alcα35 and p3-Alcβ37, showed notably better viability in the presence of p3-Alcβ37 than in the absence of it [41]. In another study, it was shown that subjects with presenilin gene mutations showed notably lower CSF p3-Alcβ levels. Other species, such as p3-Alcβ37 and p3-Alcβ40, were also decreased in the CSF of AD patients compared to age-matched controls. Such decreases in the CSF may be explained by the reduction in Alcβ expression in the brain. Taking those findings together, a decrease in p3-Alcβ in vivo may be an indicator of an increased production of Aβ42 in the brain, facilitating neuronal toxicity [42]. It has also been revealed that the Alc family proteins are cleaved by APP α-secretases (ADAM10 and ADAM17). Subsequent cleavage of the remaining C-terminal fragments involves PS1-dependent γ-secretase, which liberates a short peptide, p3-Alc, into the cell-conditioned medium and CSF. The p3-Alcα ratio of its minor to major form (p3-Alcα38/p3-Alcα35) correlated with the Aβ42/Aβ40 ratio in sporadic AD. Additionally, it has been found that alterations in the C-termini of p3-Alcs were associated with various FAD-linked PS1 mutations, proving that γ-secretase dysfunction alters the cleavage of Alc species and APP. Thus, APP and Alc are likely to be metabolized in a coordinated fashion [43].

Alcs are successively cleaved by α- and γ-secretases, leading to the release of soluble Alc ectodomains along with non-aggregative p3-Alc. Several studies suggested that changes in p3-Alc species in both CSF and blood may reflect the pathological process of Aβ accumulation. Kamogawa et al. developed a p3-Alcα35-specific ELISA, a product of γ-secretase cleavage, along with other p3-Alcα species. It was found that plasma p3-Alcα levels were increased in MCI patients and to a higher extent in AD patients compared to controls. Moreover, there was a significant positive correlation between age and plasma p3-Alcα levels in AD patients compared with normal subjects. Those findings suggest that plasma p3-Alcα levels are independently associated with AD, making it a promising novel biomarker for the presence of AD, especially at very early clinical or even preclinical stages of the disease [44]. Omori et al. found that p3-Alcα35 is the major species of the p3-Alcα peptides in plasma. Moreover, plasma p3-Alcα35 levels were increased among AD patients as well as those with lower MMSE scores compared to controls. The authors also investigated whether the levels of p3-Alcα35 changed after donepezil administration. It was shown that among AD subjects with clinical dementia rating (CDR) 1 and CDR 2, non-treated patients had significantly higher levels of plasma p3-Alcα35 when compared to levels detected in patients treated with donepezil. Thus, an increase in p3-Alcα levels may be slowed by the suppression of cognitive impairment through the donepezil intake [45]. Due to the fact that p3-Alcα, unlike Aβ and p3 of APP, is a non-aggregable and stable peptide, changes in the CSF and plasma are expected to be a marker for assessing the alteration in substrate cleavage by γ-secretase. Table 1 presents the mean values of alcadein species in CSF and plasma.

### 3.3. Furin

Furin is a proprotein convertase, catalyzing the maturation of many prohormones and proproteins in the secretory pathway compartments. There is a huge variety of substrates of furin in the brain, such as the brain-derived neurotrophic factor (BDNF), nerve-growth factor (NGF), α- and β-secretases, the matrix metalloproteinases, and other enzymes and receptors. The cleavage and release of furin’s propeptide expose its active site, allowing it to function as an endoprotease and process its substrates [47]. A growing body of research points to the fact that furin is involved in the amyloidogenic processing of APP, as BACE1 requires proteolytic removal of its proregion by furin. Bennett et al. showed that the BACE propeptide may be cleaved by the calcium-dependent protease in the Golgi apparatus, such as furin. Moreover, furin and BACE hemagglutinin epitope tag (BACE-HA) largely co-localize within shared intracellular compartments. Recombinant furin cleaves purified, soluble BACE-HA at Glu-46 in vitro, which corresponds to the known starting point of mature BACE. Furthermore, in furin-deficient cells, the cleavage of the BACE propeptide is inhibited, and furin transfection rescues this cleavage [48]. Research conducted by Hwang et al. revealed that there is a decreased expression of furin mRNA in the brains of AD patients and in a Tg2576 AD mouse model. Additionally, reduced expression was observed in the cortices of 4- and 24-month-old mice, suggesting that the reduction in furin takes place at an early age, prior to Aβ plaque formation. The authors also found that after injecting the furin-adenovirus into transgenic Tg2576 mouse brains, the production of Aβ in the infected brain regions was significantly reduced. A possible explanation could be the increase in α-secretase activity by furin cleavage of ADAM10 and tumor necrosis factor-α-converting enzyme (TACE) [49]. A similar study indicated a decrease in furin level in the cortex of AD mice. Furthermore, increased furin expression in the mouse brain enhanced BDNF maturation and promoted dendritic spine density and memory in transgenic mice [50].

A growing body of research points to the fact that the expression of furin is inhibited by iron overload [51,52]. Zhang et al. evaluated changes in furin expression in the hippocampus, an area of the brain closely associated with cognitive function. A significant decrease in furin mRNA levels was observed among APP/PS1 mice. Moreover, there was an increase in iron levels in the hippocampus. Such coexistence of furin downregulation with iron overload may suggest that these are involved in the pathogenesis of AD. Moreover, the iron accumulation via furin reduction led to a decrease in the mature BDNF, resulting in impaired formation of synapses and disturbances in learning and memory. Thus, the question was raised as to whether upregulation of furin would improve synaptic and cognitive function. Indeed, the mice with overexpression of furin alone and furin and BDNF combined exhibited better memory performance, measured by the Morris water maze tests. Therefore, furin may have potential as a therapeutic target for neurodegenerative diseases that are accompanied by iron overload [53].

### 3.4. Hypoxia-Inducible Factor-1

Hypoxia is characterized by a deficiency of oxygen in tissues. Since the brain requires a constant supply of oxygen, any disruption may lead to significant and potentially damaging effects on its function. Moreover, the evidence shows that reduced oxygen supply has been observed in the AD pathology [54]. Hypoxia-inducible factor-1 (HIF-1) is a heterodimeric transcription factor that binds DNA and plays a central role in regulating cellular responses to low oxygen levels, supporting cell survival and quick adaptation under hypoxic conditions. It is composed of an oxygen-sensitive α subunit and a stably expressed β subunit. Three main types of HIF-1 may be distinguished: HIF-1α, HIF-2α, and HIF-3α. HIF-1α mediates the acute response to hypoxia, including regulation of erythropoietin, while HIF-2α is primarily involved in the adaptation to prolonged hypoxic conditions. In the condition of low oxygen level, HIF-1α, as a hypoxia-induced nuclear transcription factor, regulates the adaptation of cells. In the brain tissue, HIF-1α is activated during hypoxia and ischemia [55].

It has been revealed that people who have suffered from severe hypoxia or ischemia are at higher risk of AD [56,57,58]. Moreover, typical changes in AD pathology, such as Aβ accumulation, abnormal phosphorylation of the tau protein, and neuronal death, are possible mechanisms of hypoxia, leading to the development of AD [59]. Tarkowska et al. found that hypoxia during the perinatal period may be a causative factor for the onset of AD later in adult life [60]. It has been shown that hypoxia has an impact on the formation and metabolism of Aβ. Specifically, research indicates that hypoxia decreases the expression and activity of ADAM 10 and ADAM 17 in neurons, therefore lowering the cleavage of APP via the non-amyloidogenic pathway. As a consequence, APP processing is shifted to the amyloidogenic pathway, resulting in a lack of sAPPα, which is known to protect against ischemic damage and glutamate neurotoxicity [61]. Auerbach et al. found that hypoxia decreases the mature form of ADAM 10 and increases the immature form, thus reducing the α-secretase processing of APP [62]. Furthermore, studies found that chronic hypoxia enhances the expression of BACE1, facilitating the amyloidogenic pathway of APP [63]. Guglielmotto et al. performed in vitro and in vivo studies and revealed that hypoxia upregulates the expression of BACE1 through a biphasic mechanism. Moreover, they found that early post-hypoxic upregulation is dependent on the release of reactive oxygen species (ROS) from mitochondria, while the late expression of BACE1 is attributed to the activation of HIF-1α [64]. Zhang et al. observed reduced BACE1 expression in the hippocampus and cortex of HIF-1α-deficient mice, proving that there is an evident link between hypoxia and APP processing, and thus Aβ production. The study also showed that Aβ generation was elevated, accompanied by an increased endogenous BACE1 level upon hypoxia treatment, suggesting a correlation between the generation of Aβ and an increase in BACE1 [65].

HIF-1α is also able to bind to and activate γ-secretase. Alexander et al. revealed that HIF-1α binds to γ-secretase, elevating the amount of active γ-secretase complex without affecting the level of individual subunits in the hypoxic–ischemic mouse brain. Moreover, the expression of full-length HIF-1α increases the activity of both BACE1 and γ-secretase in primary neuron culture. However, contrary to BACE1, HIF-1α activates γ-secretase for Aβ production in a non-transcriptional manner. Thus, preventing the binding of HIF-1α to γ-secretase would allow for the sole inhibition of γ-secretase activity during hypoxia, specifically decreasing Aβ production [66]. Due to the fact that the exact pathogenesis of AD is still unknown, the relationship between hypoxia and AD may help in gaining more knowledge about the disease and thus help develop potential therapeutic approaches.

### 3.5. Cellular Prion Protein

The cellular prion protein (PrP^c^) is a highly conserved protein, attached to the outer surface of the cell membrane by a GPI anchor. Membrane-anchored PrP^c^ undergoes internalization and recycling through the endosomal pathway, ultimately being degraded in lysosomes or released into the extracellular space [67]. It is particularly enriched in synaptic regions, as well as in astrocytes and oligodendrocytes. The highest concentrations were found in the hippocampus, cerebellum, and cerebral cortex. PrP^c^ is involved in a variety of processes in the nervous system, such as neuronal transmission, synaptic plasticity, neurite outgrowth, circadian rhythm, memory, and motor behavior [68]. Although PrP^c^ is mainly associated with prion diseases, where it aggregates to its pathogenic form, scrapie prion protein (PrP^Sc^), it has been found to also mediate the toxic effects of Aβ oligomers to some extent; thus, attention has been directed towards the role of PrP^c^ in the pathophysiology of AD. Genetic studies have revealed that the PRNP gene, which encodes PrP^c^, is a potential AD susceptibility gene, and that the Met/Val polymorphism at residue 129 in PrP^c^ has been reported as a risk factor for EOAD [69].

Whitehouse et al. demonstrated that PrP^c^ was significantly decreased in sporadic AD by a mean of 49% compared to controls. However, such a decrease may not be explained by age, as there were no notable differences between sporadic AD and control cases, suggesting that the reduction in PrP^c^ may not be attributed to the effects of age. Additionally, there were no notable differences in the level of the neuron-specific enolase (NSE) between AD and controls, which excludes the possibility of decreased PrP^c^ due to neuronal loss. Furthermore, an inverse correlation between PrP^c^ and BACE1 activity and between PrP^c^ and Aβ load in the cortex of patients with sporadic AD was observed. Finally, an inverse correlation between PrP^c^ and Braak stage was noted [70]. Parkin et al. conducted a study to verify the interaction between PrP^c^ and APP and showed that the cellular overexpression of PrP^c^ inhibited the cleavage of APP by BACE1 and thus reduced Aβ formation. Furthermore, such an effect of PrP^c^ on BACE1 required the localization of PrP^c^ to cholesterol-rich lipid rafts and was mediated by the N-terminal polybasic region of PrP^c^ via the interaction with glycosaminoglycans (GAGs). Additionally, in the brains of four-week-old mice lacking PrP^c^, the amounts of both Aβ40 and Aβ42 were notably increased compared to control, wild-type mice, proving the inhibitory effect of PrP^c^ on BACE1 [71]. Moreover, the incubation of cells with heparin resulted in the reversed inhibitory effect of PrP^c^ on BACE1-dependent APP cleavage. Taking this together, the possible mechanism of BACE1 inhibition by PrP^c^ may be by an interaction of the PrP^c^ N-terminus, through GAGs, with one or more of the heparin-binding sites on BACE1, causing a restricted access of BACE1 to APP [71]. APP/PS1 Tg mice, treated for 2 weeks with injections of 6D11 anti-PrP antibodies, showed improved performance in cognitive learning tasks and behaved the same as wild-type mice. The authors attributed this to the blocking of Aβ oligomer binding to PrP^c^, which is consistent with the critical role of PrP^c^ for mediating Aβ oligomer toxicity [72].

### 3.6. α-Synuclein

α-synuclein (α-Syn) is an abundant neuronal protein that maintains a balance between soluble and membrane-associated forms. Soluble α-Syn is unstructured and monomeric. Upon binding to highly curved membranes, like those of synaptic vesicles, α-Syn goes through conformational changes, folding into an amphipathic α-helix that promotes multimerization and facilitates its role in SNARE-complex chaperoning. In pathological conditions, soluble α-Syn forms β-sheet-like oligomers that convert into amyloid fibrils and deposit into Lewy bodies [73]. Given the fact that Aβ and α-Syn pathology overlap in some dementia disorders, more and more research aims to investigate this relationship. Both proteins are expressed abundantly in synapses and have been implicated in neuronal plasticity, learning, and memory [74]. Masliah et al. generated transgenic mice with neuronal expression of both Aβ and α-Syn. It turned out that these mice had impairment in learning and memory, developed motor deficits, and exhibited degeneration of cholinergic neurons and presynaptic terminals. Additionally, more α-Syn neuronal inclusions were observed in double-transgenic mice compared to single-transgenic ones (α-syn only). Thus, Aβ peptides promoted the aggregation of α-Syn and its intraneuronal accumulation. Interestingly, the functional and morphological patterns in the double-transgenic mice model mirrored changes occurring in dementia with Lewy bodies [75].

Roberts et al. investigated the amyloidogenic processing of APP in α-Syn-overexpressing SH-SY5Y cells. It was shown that there is an increase in amyloidogenic processing in cells overexpressing α-Syn. To verify whether it was due to an increase in BACE1 or γ-secretase, or to the increased colocalization with APP, the authors measured C99, which indicates the C-terminal fragment produced by β-cleavage. The results showed that C99 was increased by approximately 60%, suggested by a higher ratio of βCTF to full-length APP. Additionally, there were no significant changes after γ-secretase measurement. Furthermore, enhanced BACE1-mediated processing of APP corresponded with an increase in BACE1 expression in human neuroblastoma cells. In contrast, BACE1 promoter activity was reduced by α-Syn overexpression. Thus, β-secretase-mediated processing of APP may be potentiated by high levels of α-Syn and could represent a contributing mechanism of Aβ accumulation [76].

## 4. Conclusions

**Figure 2:**
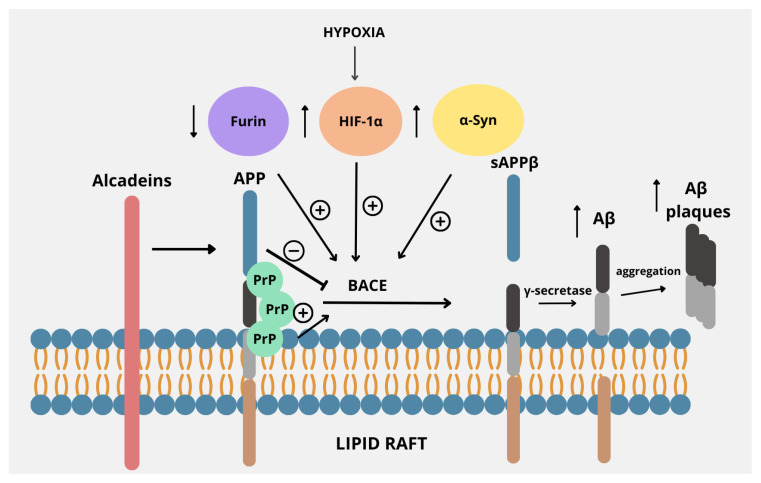
Regulators of the amyloidogenic pathway in APP processing. APP is located within the lipid raft in the plasma membrane, proving that lipid rafts are a common environment for APP processing by BACE. Its activity is regulated by several factors. Decreased levels of furin, often accompanied by iron overload, stimulate the amyloidogenic pathway of APP processing by increasing the activity of BACE. Also, factors such as increased α-synuclein or HIF-1α expression due to hypoxia cause upregulation of BACE. Alcadeins act more indirectly, as these proteins exert neuroprotective functions by preventing cleavage by BACE, thus causing a shift from the amyloidogenic to non-amyloidogenic pathway. PrP, by binding with APP within lipid rafts, enhances the activity of BACE, thus promoting the amyloidogenic pathway.

A mounting body of evidence points to the fact that aberrant processing of APP, resulting in the release of toxic Aβ peptides, is a central point in the development of AD. Aberrant processing of the β- and γ-secretases may lead to an imbalance between the production and the clearance of Aβ peptides, resulting in the formation of toxic oligomers, fibrils, and senile plaques. Thus, the regulators of such processes may be crucial in understanding the exact mechanisms and introducing new potential therapies [77]. Figure 2 presents the main points of interaction between APP and the proteins described in this review.

In addition to their normal functions, including membrane trafficking, ligand binding, axonal development, and maintenance of synaptic integrity, lipid rafts have been implicated in the pathogenesis of AD, as they promote the interaction of APP with BACE-1, which is responsible for the generation of the Aβ peptides [78]. Alcadeins, particularly Alc-α, modulate the amyloidogenic processing of APP by competing for the same secretases and interacting with trafficking proteins like X11/Mint. This competition and interaction help retain APP in the trans-Golgi network, keeping it away from endosomes, where β-secretase is active. As a result, alcadeins reduce Aβ production and favor non-amyloidogenic processing, making them potential modulators of AD pathology [79]. Furin promotes the non-amyloidogenic pathway by enhancing sAPPα production, which in turn reinforces this processing route. Inhibition of furin reduces sAPPα levels—without altering APP levels—indicating a shift toward amyloidogenic processing [49]. Concerning HIF-1α, findings collectively revealed that there is an evident link between hypoxia or ischemia and APP expression and processing that may contribute to AD pathogenesis. It has been shown that hypoxia increases *BACE1* gene transcription at mRNA levels through the induction of HIF-1 with the resultant increased BACE activity and Aβ production [80]. PrP^c^ also plays an important role in the pathophysiology of AD, especially considering the fact that the PrP^c^ deposits often accompany Aβ plaques in AD. The interaction between these two proteins was even more evident after demonstrating that PrP^c^ exhibits high affinity to oligomeric Aβ. It suggests that it is important for Aβ-induced neurotoxicity, as demonstrated by the evident loss of LTP and memory impairment in AD mouse models [81]. Moreover, deficiency of PrP^c^ confers resistance to the synaptic toxicity of oligomeric Aβ in mice and in hippocampal slice cultures in vitro [82]. Thus, the interaction between PrP^c^ and Aβ is crucial for neurotoxicity and neuronal cell loss in AD. Finally, α-Syn, primarily associated with Parkinson’s disease, has been shown to be deeply involved in the pathogenesis of AD, especially since a significant proportion of AD subjects have underlying α-Syn pathology. Indeed, studies have shown that the BACE1-mediated processing of APP may be potentiated by high levels of α-Syn; thus, understanding their multifaceted interactions could provide insights into the pathophysiology of AD and highlight potential therapeutic avenues [76,83].

The regulatory factors discussed in this review represent promising targets for the therapeutic modulation of APP processing. Several of the discussed APP processing regulators also hold potential as biomarkers for early AD diagnosis. These biomarkers, if validated in longitudinal cohorts, could improve early detection and risk stratification in preclinical AD stages.

Cholesterol-rich lipid rafts provide a scaffold that brings APP and secretases into close proximity, thereby promoting amyloidogenic processing [84]. Analyzing lipid raft distribution across various brain regions may be crucial for detecting early neurodegenerative changes, even before clinical symptoms emerge. Precise quantification of raft-associated components—such as caveolin-1, IGF-1R, BDNF, GPI-anchored proteins, and their by-products—in CSF or blood, combined with multivariate methods like discriminant function analysis, could serve as a valuable diagnostic tool for more accurate detection and monitoring of Alzheimer’s disease [85]. While emerging evidence highlights furin’s diagnostic and functional relevance in AD, no interventional trials targeting its activity have yet been launched. CSF or plasma furin measurements could be pursued in longitudinal cohorts to validate its predictive or diagnostic value. Longitudinal studies are needed to validate its predictive value and establish standardized measurement protocols. Ultimately, furin could complement established markers and contribute to early diagnosis, staging, and response assessment in AD [86]. Alcadeins also hold potential as both biomarkers and therapeutic targets in Alzheimer’s disease (AD), although research in this area is still emerging and largely preclinical. Specifically, elevated plasma p3-Alcα, particularly in the early stages of cognitive decline, suggests that Alc metabolites are useful plasma biomarkers of AD. Furthermore, enhancing Alcα function or expression may shift APP processing toward the non-amyloidogenic pathway, reducing Aβ generation. However, no current clinical trials specifically target Alcs, and further studies are required to validate their clinical utility [41,42,43,44,45]. Given its role in the key pathological processes of Alzheimer’s disease—including cerebral hypoperfusion, neuroinflammation, and impaired synaptic plasticity—HIF-1α emerges as a promising therapeutic target. Modulating its activity may open new avenues for preventing neurodegeneration and preserving cognitive function in AD patients [87]. The previously discussed findings indicate that therapeutics targeting PrPC may be most effectively evaluated in cases of mild to moderate AD, where they are likely to exert a rapid symptomatic impact on cognition and memory. Moreover, PrPC facilitates the formation of specific Aβ oligomeric species at the synapse and may also contribute to the toxicity of other β-sheet-rich oligomers. In mice, therapeutic strategies employing the soluble PrPC ectodomain or monoclonal antibodies directed against PrPC have shown potential in partially mitigating the neurotoxic effects of Aβ oligomers [88]. Additionally, recent research has shown that miR-519a-3p has been implicated in the downregulation of PrPC during AD progression. Its early expression in the disease course makes it a promising candidate for inclusion in an asymptomatic, AD-specific molecular signature, with strong potential as a blood-based biomarker warranting further investigation [89]. α-Syn may potentiate APP amyloidogenic processing by upregulating BACE1 expression or activity, thus increasing Aβ production. It can also contribute to synaptic dysfunction and neuroinflammation in AD. Therefore, inhibiting α-synuclein aggregation, enhancing its clearance, or blocking its interaction with BACE1 or tau are therapeutic approaches being explored in models of AD [90].

The identification of novel regulators of amyloidogenic APP processing has significant implications for the development of both diagnostic tools and therapeutic interventions. The molecular targets highlighted here may be incorporated into future high-throughput drug screening platforms or serve as inclusion criteria for patient stratification in clinical trials. The integration of these findings into early-phase clinical research may accelerate the development of precision therapies for Alzheimer’s disease.

To conclude, regulators of the APP amyloidogenic pathway are crucial in the development and progression of AD. Understanding the complex interplay of these regulators provides insights into novel approaches for diagnosing and managing AD.

## Figures and Tables

**Table 1:** Mean values of different alcadein species in CSF and plasma.

Protein	Group	Mean Value	Method	Source
CSF				
p3-Alcβ37	Patients along the AD continuum		ELISA	[41]
	A−	3928 pg/mL		
	A+T−N−	1889 pg/mL		
	A+T+N−	2899 pg/mL		
	A+T+N+	3808 pg/mL		
p3-Alcβ37	I cohort:		ELISA	[42]
	controls vs. AD	9394 pg/mL vs. 6614 pg/mL		
	controls vs. MCI	9394 pg/mL vs. 9596 pg/mL		
	MCI vs. AD	9596 pg/mL vs. 6614 pg/mL		
	II cohort:			
	controls vs. AD	6421 pg/mL vs. 4780 pg/mL		
	controls vs. MCI	6421 pg/mL vs. 6296 pg/mL		
	MCI vs. AD	6296 pg/mL vs. 4780 pg/mL		
p3-Alcβ40	I cohort:		ELISA	[42]
	controls vs. AD	1212 pg/mL vs. 884.1 pg/mL		
	controls vs. MCI	1212 pg/mL vs. 1177 pg/mL		
	MCI vs. AD	1177 pg/mL vs. 884.1 pg/mL		
	II cohort:			
	controls vs. AD	1032 pg/mL vs. 931.5 pg/mL		
	controls vs. MCI	1032 pg/mL vs. 1065 pg/mL		
	MCI vs. AD	1065 pg/mL vs. 931.5 pg/mL		
	III cohort:			
	controls vs. AD	651.4 pg/mL vs. 509.8 pg/mL		
	controls vs. MCI	509.8 pg/mL vs. 544.1 pg/mL		
	MCI vs. AD	544.1 pg/mL vs. 509.8 pg/mL		
Plasma				
p3-Alcα35	controls vs. AD	189.1 pg/mL vs. 224.7 pg/mL	ELISA	[44]
	controls vs. MCI	189.1 pg/mL vs. 223.3 pg/mL		
	MCI vs. AD	223.3 pg/mL vs. 224.7 pg/mL		
p3-Alcα35	I cohort:		ELISA	[45]
	controls vs. AD	140.1 pg/mL vs. 178.7 pg/mL		
	controls vs. MCI	140.1 pg/mL vs. 166.3 pg/mL		
	MCI vs. AD	166.3 pg/mL vs. 178.7 pg/mL		
	II cohort:			
	controls vs. AD	148 pg/mL vs. 196.5 pg/mL		
	controls vs. MCI	148 pg/mL vs. 166.4 pg/mL		
	MCI vs. AD	166.4 pg/mL vs. 196.5 pg/mL		
	III cohort:			
	controls vs. AD	128.6 pg/mL vs. 130.8 pg/mL		
	controls vs. MCI	128.6 pg/mL vs. 146.0 pg/mL		
	MCI vs. AD	146.0 pg/mL vs. 130.8 pg/mL		
total p3-Alcα	controls vs. AD	163 pg/mL vs. 232 pg/mL	ELISA	[46]

## Data Availability

No new data were created or analyzed in this study.

## References

[1] Wong W. (2020). Economic burden of Alzheimer disease and managed care considerations. Am. J. Manag. Care.

[2] Rostagno A.A. (2023). Pathogenesis of Alzheimer’s Disease. Int. J. Mol. Sci..

[3] Aisen P.S., Cummings J., Jack C.R., Morris J.C., Sperling R., Frölich L., Jones R.W., Dowsett S.A., Matthews B.R., Raskin J. (2017). On the path to 2025: Understanding the Alzheimer’s disease continuum. Alzheimer’s Res. Ther..

[4] Zvěřová M. (2019). Clinical aspects of Alzheimer’s disease. Clin. Biochem..

[5] Nasb M., Tao W., Chen N. (2024). Alzheimer’s Disease Puzzle: Delving into Pathogenesis Hypotheses. Aging Dis..

[6] Krishnamurthy H.K., Jayaraman V., Krishna K., Wang T., Bei K., Changalath C., Rajasekaran J.J. (2025). An overview of the genes and biomarkers in Alzheimer’s disease. Ageing Res. Rev..

[7] Orobets K.S., Karamyshev A.L. (2023). Amyloid Precursor Protein and Alzheimer’s Disease. Int. J. Mol. Sci..

[8] Al-Kuraishy H.M., Jabir M.S., Al-Gareeb A.I., Albuhadily A.K., Albukhaty S., Sulaiman G.M., Batiha G.E. (2023). Evaluation and targeting of amyloid precursor protein (APP)/amyloid beta (Aβ) axis in amyloidogenic and non-amyloidogenic pathways: A time outside the tunnel. Ageing Res. Rev..

[9] Young-Pearse T.L., Chen A.C., Chang R., Marquez C., Selkoe D.J. (2008). Secreted APP regulates the function of full-length APP in neurite outgrowth through interaction with integrin β1. Neural Dev..

[10] Hefter D., Draguhn A. (2017). APP as a Protective Factor in Acute Neuronal Insults. Front. Mol. Neurosci..

[11] Obregon D., Hou H., Deng J., Giunta B., Tian J., Darlington D., Shahaduzzaman M., Zhu Y., Mori T., Mattson M.P. (2012). Soluble amyloid precursor protein-α modulates β-secretase activity and amyloid-β generation. Nat. Commun..

[12] Taylor H.A., Przemylska L., Clavane E.M., Meakin P.J. (2022). BACE1: More than just a β-secretase. Obes. Rev..

[13] Kamenetz F., Tomita T., Hsieh H., Seabrook G., Borchelt D., Iwatsubo T., Sisodia S., Malinow R. (2003). APP processing and synaptic function. Neuron.

[14] Zhang Z., Song M., Liu X., Su Kang S., Duong D.M., Seyfried N.T., Cao X., Cheng L., Sun Y.E., Ping Yu S. (2015). Delta-secretase cleaves amyloid precursor protein and regulates the pathogenesis in Alzheimer’s disease. Nat. Commun..

[15] Wang S.S., Liu Z.K., Liu J.J., Cheng Q., Wang Y.X., Liu Y., Ni W.W., Chen H.Z., Song M. (2021). Imaging asparaginyl endopeptidase (AEP) in the live brain as a biomarker for Alzheimer’s disease. J. Nanobiotechnol..

[16] Afram E., Lauritzen I., Bourgeois A., El Manaa W., Duplan E., Chami M., Valverde A., Charlotte B., Pardossi-Piquard R., Checler F. (2023). The η-secretase-derived APP fragment ηCTF is localized in Golgi, endosomes and extracellular vesicles and contributes to Aβ production. Cell. Mol. Life Sci..

[17] Willem M., Tahirovic S., Busche M.A., Ovsepian S.V., Chafai M., Kootar S., Hornburg D., Evans L.D., Moore S., Daria A. (2015). η-Secretase processing of APP inhibits neuronal activity in the hippocampus. Nature.

[18] Pilat D., Paumier J.M., Louis L., Manrique C., García-González L., Stephan D., Bernard A., Pardossi-Piquard R., Checler F., Khrestchatisky M. (2024). Suppression of MT5-MMP Reveals Early Modulation of Alzheimer’s Pathogenic Events in Primary Neuronal Cultures of 5xFAD Mice. Biomolecules.

[19] Fu J., Lai X., Huang Y., Bao T., Yang J., Chen S., Chen X., Shang H. (2023). Meta-analysis and systematic review of peripheral platelet-associated biomarkers to explore the pathophysiology of Alzheimer’s disease. BMC Neurol..

[20] Li T.R., Liu F.Q. (2022). β-Amyloid promotes platelet activation and activated platelets act as bridge between risk factors and Alzheimer’s disease. Mech. Ageing Dev..

[21] Al-Kuraishy H.M., Sulaiman G.M., Mohammed H.A., Dawood R.A., Albuhadily A.K., Al-Gareeb A.I., Abomughaid M.M., Klionsky D.J. (2025). Alterations in the Processing of Platelet APP (Amyloid Beta Precursor Protein) in Alzheimer Disease: The Possible Nexus. Neuropsychopharmacol. Rep..

[22] Ramos-Cejudo J., Johnson A.D., Beiser A., Seshadri S., Salinas J., Berger J.S., Fillmore N.R., Do N., Zheng C., Kovbasyuk Z. (2022). Platelet Function Is Associated with Dementia Risk in the Framingham Heart Study. J. Am. Heart Assoc..

[23] Burrinha T., Guimas Almeida C. (2022). Aging impact on amyloid precursor protein neuronal trafficking. Curr. Opin. Neurobiol..

[24] Sasmita A.O., Depp C., Nazarenko T., Sun T., Siems S.B., Ong E.C., Nkeh Y.B., Böhler C., Yu X., Bues B. (2024). Oligodendrocytes produce amyloid-β and contribute to plaque formation alongside neurons in Alzheimer’s disease model mice. Nat. Neurosci..

[25] Wessels A.M., Lines C., Stern R.A., Kost J., Voss T., Mozley L.H., Furtek C., Mukai Y., Aisen P.S., Cummings J.L. (2020). Cognitive outcomes in trials of two BACE inhibitors in Alzheimer’s disease. Alzheimer’s Dement..

[26] Dunot J., Ribera A., Pousinha P.A., Marie H. (2023). Spatiotemporal insights of APP function. Curr. Opin. Neurobiol..

[27] Kao Y.C., Ho P.C., Tu Y.K., Jou I.M., Tsai K.J. (2020). Lipids and Alzheimer’s Disease. Int. J. Mol. Sci..

[28] Arbor S.C., LaFontaine M., Cumbay M. (2016). Amyloid-beta Alzheimer targets—Protein processing, lipid rafts, and amyloid-beta pores. Yale J. Biol. Med..

[29] Cheng H., Vetrivel K.S., Gong P., Meckler X., Parent A., Thinakaran G. (2007). Mechanisms of disease: New therapeutic strategies for Alzheimer’s disease—Targeting APP processing in lipid rafts. Nat. Clin. Pract. Neurol..

[30] Cordy J.M., Hussain I., Dingwall C., Hooper N.M., Turner A.J. (2003). Exclusively targeting β-secretase to lipid rafts by GPI-anchor addition up-regulates β-site processing of the amyloid precursor protein. Proc. Natl. Acad. Sci. USA.

[31] Ehehalt R., Keller P., Haass C., Thiele C., Simons K. (2003). Amyloidogenic processing of the Alzheimer β-amyloid precursor protein depends on lipid rafts. J. Cell Biol..

[32] Díaz M., Fabelo N., Martín V., Ferrer I., Gómez T., Marín R. (2015). Biophysical alterations in lipid rafts from human cerebral cortex associate with increased BACE1/AβPP interaction in early stages of Alzheimer’s disease. J. Alzheimer’s Dis..

[33] Fabelo N., Martín V., Marín R., Moreno D., Ferrer I., Díaz M. (2014). Altered lipid composition in cortical lipid rafts occurs at early stages of sporadic Alzheimer’s disease and facilitates APP/BACE1 interactions. Neurobiol. Aging.

[34] Blaskovic S., Blanc M., van der Goot F.G. (2013). What does S-palmitoylation do to membrane proteins?. FEBS J..

[35] Smotrys J.E., Linder M.E. (2004). Palmitoylation of intracellular signaling proteins: Regulation and function. Annu. Rev. Biochem..

[36] Cheng H., Vetrivel K.S., Drisdel R.C., Meckler X., Gong P., Leem J.Y., Li T., Carter M., Chen Y., Nguyen P. (2009). S-palmitoylation of γ-secretase subunits nicastrin and APH-1. J. Biol. Chem..

[37] Um J.W., Pramanik G., Ko J.S., Song M.Y., Lee D., Kim H., Park K.S., Südhof T.C., Tabuchi K., Ko J. (2014). Calsyntenins function as synaptogenic adhesion molecules in concert with neurexins. Cell Rep..

[38] Piao Y., Kimura A., Urano S., Saito Y., Taru H., Yamamoto T., Hata S., Suzuki T. (2013). Mechanism of intramembrane cleavage of alcadeins by γ-secretase. PLoS ONE.

[39] Motodate R., Saito Y., Hata S., Suzuki T. (2016). Expression and localization of X11 family proteins in neurons. Brain Res..

[40] Honda K., Takahashi H., Hata S., Abe R., Saito T., Saido T.C., Taru H., Sobu Y., Ando K., Yamamoto T. (2024). Suppression of the amyloidogenic metabolism of APP and the accumulation of Aβ by alcadein α in the brain during aging. Sci. Rep..

[41] Hata S., Saito H., Kakiuchi T., Fukumoto D., Yamamoto S., Kasuga K., Kimura A., Moteki K., Abe R., Adachi S. (2023). Brain p3-Alcβ peptide restores neuronal viability impaired by Alzheimer’s amyloid β-peptide. EMBO Mol. Med..

[42] Hata S., Omori C., Kimura A., Saito H., Kimura N., Gupta V., Pedrini S., Hone E., Chatterjee P., Taddei K. (2019). Decrease in p3-Alcβ37 and p3-Alcβ40, products of Alcadein β generated by γ-secretase cleavages, in aged monkeys and patients with Alzheimer’s disease. Alzheimer’s Dement..

[43] Hata S., Fujishige S., Araki Y., Kato N., Araseki M., Nishimura M., Hartmann D., Saftig P., Fahrenholz F., Taniguchi M. (2009). Alcadein cleavages by amyloid β-precursor protein (APP) α- and γ-secretases generate small peptides, p3-Alcs, indicating Alzheimer disease-related γ-secretase dysfunction. J. Biol. Chem..

[44] Kamogawa K., Kohara K., Tabara Y., Takita R., Miki T., Konno T., Hata S., Suzuki T. (2012). Potential utility of soluble p3-alcadein α plasma levels as a biomarker for sporadic Alzheimer’s disease. J. Alzheimer’s Dis..

[45] Omori C., Kaneko M., Nakajima E., Akatsu H., Waragai M., Maeda M., Morishima-Kawashima M., Saito Y., Nakaya T., Taru H. (2014). Japanese Alzheimer’s Disease Neuroimaging Initiative. Increased levels of plasma p3-alcα35, a major fragment of Alcadeinα by γ-secretase cleavage, in Alzheimer’s disease. J. Alzheimer’s Dis..

[46] Konno T., Hata S., Hamada Y., Horikoshi-Sakuraba Y., Nakaya T., Saito Y., Yamamoto T., Yamamoto T., Maeda M., Ikeuchi T. (2011). Japanese Alzheimer’s Disease Neuroimaging Initiative. Coordinated increase of γ-secretase reaction products in the plasma of some female Japanese sporadic Alzheimer’s disease patients: Quantitative analysis of p3-Alcα with a new ELISA system. Mol. Neurodegener..

[47] Thomas G. (2002). Furin at the cutting edge: From protein traffic to embryogenesis and disease. Nat. Rev. Mol. Cell Biol..

[48] Bennett B.D., Denis P., Haniu M., Teplow D.B., Kahn S., Louis J.C., Citron M., Vassar R. (2000). A furin-like convertase mediates propeptide cleavage of BACE, the Alzheimer’s β-secretase. J. Biol. Chem..

[49] Hwang E.M., Kim S.K., Sohn J.H., Lee J.Y., Kim Y., Kim Y.S., Mook-Jung I. (2006). Furin is an endogenous regulator of α-secretase associated APP processing. Biochem. Biophys. Res. Commun..

[50] Yang Y., He M., Tian X., Guo Y., Liu F., Li Y., Zhang H., Lu X., Xu D., Zhou R. (2018). Transgenic overexpression of furin increases epileptic susceptibility. Cell Death Dis..

[51] Li J., Ding Y., Zhang J., Zhang Y., Cui Y., Zhang Y., Chang S., Chang Y.Z., Gao G. (2024). Iron overload suppresses hippocampal neurogenesis in adult mice: Implication for iron dysregulation-linked neurological diseases. CNS Neurosci. Ther..

[52] Choi D.H., Kwon K.C., Hwang D.J., Koo J.H., Um H.S., Song H.S., Kim J.S., Jang Y., Cho J.Y. (2021). Treadmill Exercise Alleviates Brain Iron Dyshomeostasis Accelerating Neuronal Amyloid-β Production, Neuronal Cell Death, and Cognitive Impairment in Transgenic Mice Model of Alzheimer’s Disease. Mol. Neurobiol..

[53] Zhang Y., Bai X., Zhang Y., Yao S., Cui Y., You L.H., Yu P., Chang Y.Z., Gao G. (2022). Hippocampal Iron Accumulation Impairs Synapses and Memory via Suppressing Furin Expression and Downregulating BDNF Maturation. Mol. Neurobiol..

[54] Adeyemi O.S., Awakan O.J., Afolabi L.B., Rotimi D.E., Oluwayemi E., Otuechere C.A., Ibraheem O., Elebiyo T.C., Alejolowo O., Arowolo A.T. (2021). Hypoxia and the Kynurenine Pathway: Implications and Therapeutic Prospects in Alzheimer’s Disease. Oxid. Med. Cell. Longev..

[55] Wang Y.Y., Huang Z.T., Yuan M.H., Jing F., Cai R.L., Zou Q., Pu Y.S., Wang S.Y., Chen F., Yi W.M. (2021). Role of Hypoxia Inducible Factor-1α in Alzheimer’s Disease. J. Alzheimers Dis..

[56] Liu G., Yang C., Wang X., Chen X., Wang Y., Le W. (2023). Oxygen metabolism abnormality and Alzheimer’s disease: An update. Redox Biol..

[57] Tao B., Gong W., Xu C., Ma Z., Mei J., Chen M. (2024). The relationship between hypoxia and Alzheimer’s disease: An updated review. Front. Aging Neurosci..

[58] Elman-Shina K., Efrati S. (2022). Ischemia as a common trigger for Alzheimer’s disease. Front. Aging Neurosci..

[59] Desmond D.W., Moroney J.T., Sano M., Stern Y. (2002). Incidence of dementia after ischemic stroke: Results of a longitudinal study. Stroke.

[60] Tarkowska A. (2021). Hypoxic-Ischemic Brain Injury after Perinatal Asphyxia as a Possible Factor in the Pathology of Alzheimer’s Disease. Cerebral Ischemia.

[61] Sun M., Zhou T., Zhou L., Chen Q., Yu Y., Yang H., Zhong K., Zhang X., Xu F., Cai S. (2012). Formononetin protects neurons against hypoxia-induced cytotoxicity through upregulation of ADAM10 and sAβPPα. J. Alzheimer’s Dis..

[62] Auerbach I.D., Vinters H.V. (2006). Effects of anoxia and hypoxia on amyloid precursor protein processing in cerebral microvascular smooth muscle cells. J. Neuropathol. Exp. Neurol..

[63] Lall R., Mohammed R., Ojha U. (2019). What are the links between hypoxia and Alzheimer’s disease?. Neuropsychiatr. Dis. Treat..

[64] Guglielmotto M., Aragno M., Autelli R., Giliberto L., Novo E., Colombatto S., Danni O., Parola M., Smith M.A., Perry G. (2009). The up regulation of BACE1 mediated by hypoxia and ischemic injury: Role of oxidative stress and HIF1α. J. Neurochem..

[65] Zhang X., Zhou K., Wang R., Cui J., Lipton S.A., Liao F.F., Xu H., Zhang Y.W. (2007). Hypoxia-inducible factor 1α (HIF-1α)-mediated hypoxia increases BACE1 expression and β-amyloid generation. J. Biol. Chem..

[66] Alexander C., Li T., Hattori Y., Chiu D., Frost G.R., Jonas L., Liu C., Anderson C.J., Wong E., Park L. (2022). Hypoxia Inducible Factor-1α binds and activates γ-secretase for Aβ production under hypoxia and cerebral hypoperfusion. Mol. Psychiatry.

[67] Chrobak A.A., Adamek D. (2014). New light on prions: Putative role of co-operation of PrPc and Aβ proteins in cognition. Folia Neuropathol..

[68] Fułek M., Hachiya N., Gachowska M., Beszłej J.A., Bartoszewska E., Kurpas D., Kurpiński T., Adamska H., Poręba R., Urban S. (2025). Cellular Prion Protein and Amyloid-β Oligomers in Alzheimer’s Disease—Are There Connections?. Int. J. Mol. Sci..

[69] Del Bo R., Scarlato M., Ghezzi S., Martinelli-Boneschi F., Fenoglio C., Galimberti G., Galbiati S., Virgilio R., Galimberti D., Ferrarese C. (2006). Is M129V of PRNP gene associated with Alzheimer’s disease? A case-control study and a meta-analysis. Neurobiol. Aging.

[70] Whitehouse I.J., Miners J.S., Glennon E.B., Kehoe P.G., Love S., Kellett K.A., Hooper N.M. (2013). Prion protein is decreased in Alzheimer’s brain and inversely correlates with BACE1 activity, amyloid-β levels and Braak stage. PLoS ONE.

[71] Parkin E.T., Watt N.T., Hussain I., Eckman E.A., Eckman C.B., Manson J.C., Baybutt H.N., Turner A.J., Hooper N.M. (2007). Cellular prion protein regulates β-secretase cleavage of the Alzheimer’s amyloid precursor protein. Proc. Natl. Acad. Sci. USA.

[72] Chung E., Ji Y., Sun Y., Kascsak R.J., Kascsak R.B., Mehta P.D., Strittmatter S.M., Wisniewski T. (2010). Anti-PrPC monoclonal antibody infusion as a novel treatment for cognitive deficits in an Alzheimer’s disease model mouse. BMC Neurosci..

[73] Burré J., Sharma M., Südhof T.C. (2018). Cell Biology and Pathophysiology of α-Synuclein. Cold Spring Harb. Perspect. Med..

[74] Clayton D.F., George J.M. (1998). The synucleins: A family of proteins involved in synaptic function, plasticity, neurodegeneration and disease. Trends Neurosci..

[75] Masliah E., Rockenstein E., Veinbergs I., Sagara Y., Mallory M., Hashimoto M., Mucke L. (2001). β-Amyloid peptides enhance α-synuclein accumulation and neuronal deficits in a transgenic mouse model linking Alzheimer’s disease and Parkinson’s disease. Proc. Natl. Acad. Sci. USA.

[76] Roberts H.L., Schneider B.L., Brown D.R. (2017). α-Synuclein increases β-amyloid secretion by promoting β-/γ-secretase processing of APP. PLoS ONE.

[77] Zhao J., Liu X., Xia W., Zhang Y., Wang C. (2020). Targeting Amyloidogenic Processing of APP in Alzheimer’s Disease. Front. Mol. Neurosci..

[78] Kalvodova L., Kahya N., Schwille P., Ehehalt R., Verkade P., Drechsel D., Simons K. (2005). Lipids as modulators of proteolytic activity of BACE: Involvement of cholesterol, glycosphingolipids, and anionic phospholipids In Vitro. J. Biol. Chem..

[79] Gotoh N., Saito Y., Hata S., Saito H., Ojima D., Murayama C., Shigeta M., Abe T., Konno D., Matsuzaki F. (2020). Amyloidogenic processing of amyloid β protein precursor (APP) is enhanced in the brains of alcadein α-deficient mice. J. Biol. Chem..

[80] Lin T.-K., Huang C.-R., Lin K.-J., Hsieh Y.-H., Chen S.-D., Lin Y.-C., Chao A.-C., Yang D.-I. (2024). Potential Roles of Hypoxia-Inducible Factor-1 in Alzheimer’s Disease: Beneficial or Detrimental?. Antioxidants.

[81] Kostylev M.A., Kaufman A.C., Nygaard H.B., Patel P., Haas L.T., Gunther E.C., Vortmeyer A., Strittmatter S.M. (2015). Prion-Protein-interacting Amyloid-β Oligomers of High Molecular Weight Are Tightly Correlated with Memory Impairment in Multiple Alzheimer Mouse Models. J. Biol. Chem..

[82] Barry A.E., Klyubin I., Mc Donald J.M., Mably A.J., Farrell M.A., Scott M., Walsh D.M., Rowan M.J. (2011). Alzheimer’s disease brain-derived amyloid-β-mediated inhibition of LTP in vivo is prevented by immunotargeting cellular prion protein. J. Neurosci..

[83] Lin S., Leitão A.D.G., Fang S., Gu Y., Barber S., Gilliard-Telefoni R., Castro A., Sung K., Shen R., Florio J.B. (2023). Overexpression of alpha synuclein disrupts APP and Endolysosomal axonal trafficking in a mouse model of synucleinopathy. Neurobiol. Dis..

[84] Cao Y., Zhao L.W., Chen Z.X., Li S.H. (2024). New insights in lipid metabolism: Potential therapeutic targets for the treatment of Alz-heimer’s disease. Front. Neurosci..

[85] Marin R., Rojo J.A., Fabelo N., Fernandez C.E., Diaz M. (2013). Lipid raft disarrangement as a result of neuro-pathological progresses: A novel strategy for early diagnosis?. Neuroscience.

[86] Zhang Y., Gao X., Bai X., Yao S., Chang Y.Z., Gao G. (2022). The emerging role of furin in neurodegenerative and neuropsychiatric diseases. Transl. Neurodegener..

[87] Porel P., Bala K., Aran K.R. (2025). Exploring the role of HIF-1α on pathogenesis in Alzheimer’s disease and potential therapeutic approaches. Inflammopharmacology.

[88] Laurén J. (2014). Cellular prion protein as a therapeutic target in Alzheimer’s disease. J. Alzheimer’s Dis..

[89] Jácome D., Cotrufo T., Andrés-Benito P., Lidón L., Martí E., Ferrer I., Del Río J.A., Gavín R. (2024). miR-519a-3p, found to regulate cellular prion protein during Alzheimer’s disease pathogenesis, as a biomarker of asymptomatic stages. Biochim. Biophys. Acta (BBA) Mol. Basis Dis..

[90] Das B., Yan R. (2019). A Close Look at BACE1 Inhibitors for Alzheimer’s Disease Treatment. CNS Drugs.

